# Field resistance of *Spodoptera litura* (Lepidoptera: Noctuidae) to organophosphates, pyrethroids, carbamates and four newer chemistry insecticides in Hunan, China

**DOI:** 10.1007/s10340-013-0505-y

**Published:** 2013-05-07

**Authors:** Hong Tong, Qi Su, Xiaomao Zhou, Lianyang Bai

**Affiliations:** 1 Institute of Pesticide Science, Hunan Agricultural University, Changsha, 410128 People’s Republic of China; 2 Hunan Institute of Humanities, Science and Technology, Loudi, 417000 People’s Republic of China

**Keywords:** Cutworm, Emamectin benzoate, Abamectin, Indoxacarb, Chlorfenapyr, Resistance evolution, Cross-resistance

## Abstract

The present studies were carried out to evaluate resistance in the populations of *Spodoptera litura* Fab. (Lepidoptera, Noctuidae) from five districts of Hunan Province in China to various insecticides from 2010 to 2012 using a standard leaf dip bioassay method. For organophosphates and pyrethroids, resistance ratios compared with a susceptible Lab-BJ strain were in the range of 14–229-fold for organophosphates and 12–227-fold for pyrethroids. Similarly, relative low levels of resistance to emamectin, indoxacarb, and chlorfenapyr were observed in all five populations. In contrast, the resistance to carbamates (thiodicarb or methomyl) was significantly higher than that of organophosphates, pyrethroids and newer chemistry insecticides. The pairwise correlation coefficients of LC_50_ values indicated that the newer chemistry insecticides and old generation insecticides were not significant except abamectin, which was negatively significantly correlated with methomyl. A significant correlation was observed between thiodicarb, methomyl, and deltamethrin, whereas resistance to bifenthrin showed no correlations with resistance to other insecticides except deltamethrin. The results are discussed in relation to integrated pest management for *S. litura* with special reference to management of field evolved resistance to insecticides.

## Introduction

The cutworm *Spodoptera litura* Fab. (Lepidoptera, Noctuidae) is well known as a serious cosmopolitan pest with extensive host range of economically important crops such as cotton, groundnut, soybean, tomato, sweet potato, and many other crops (Matsuura and Naito 1997; Sahayaraj and Paulraj 1998). *S. litura* has been shown to be resistant to a wide range of insecticides, which has led to sporadic out breaks of the pest and failure of crops (Ahmad et al. 2007a). Since the pest was found in Hunan Province of China, its damage has increased continually. Its control has depended mostly on application of various insecticides. As a result, many field populations of this pest have developed high resistance against wide variety of insecticides including organophosphate, carbamate, pyrethroids and some selected newer chemistry insecticides with field control failure observed very frequently (Armes et al. 1997; Kranthi et al. 2001; Ahmad et al. 2007a, b, 2008; Saleem et al. 2008). The management of the pest has therefore become increasingly difficult all over the world and the most commonly used insecticides are ineffective in controlling it.

Resistance to insecticides is a major problem associated with the chemical control of insect pests, which is characterized by rapid evolution under strong selection of gene(s) that confers survival to insecticides (Ahmad et al. 2008). This selective pressure exerted by the insecticides abruptly increases the frequency of the genetic condition expressed as resistance within the exposed population. The development of resistance is a result of the selection pressure exerted on sprayed populations increasing the frequency of resistant individuals (Torres-Vila et al. 2002), but several factors including temperature are also involved in influencing the evolution of insecticides resistance (Raymond and Marquine 1994). At present, the extensive use of conventional insecticides such as organophosphate, carbamate and pyrethroids against *S. litura* has produced prevalent resistance in China (Wu et al. 1995; Huang et al. 2006). With high resistance to conventional insecticides, the insect growth regulators (IGRs) and newer insecticides were recently introduced to control this pest (Chen et al. 2008; Su et al. 2012). In the case of IGRs, flufenoxuron, chlorfluazuron, tebufenozide, and methoxyfenozide were used to control *S. litura* in Shandong and Jiangsu Provinces and had high toxicity to *S. litura*, in which resistance to flufenoxuron and methoxyfenozide was barely produced (Huang et al. 2006). In addition, the newer insecticides bearing novel modes of action such as indoxacarb, abamectin, emamectin benzoate, fipronil, and spinosad were recently introduced into Hunan Province for management of the pests. The extensive use of these newer insecticides against *S. litura* have provided an ideal environment for its evolution of resistance and *S. litura* was found to have inherent risks for resistance to indoxacarb (Wang et al. 2009). Previous exposure and selection with insecticides can confer resistance to newly introduced insecticides through cross-resistance (Bisset et al. 1997; Sayyed et al. 2008), reducing the effectiveness of many new insecticides. There are some data available on the newer insecticide resistance in *S. litura* from cash crops and vegetables growing countries such as Pakistan (Ahmad et al. 2008; Shad et al. 2012).

Following reports of poor efficacy of the newer chemistry insecticides against *S. litura* both in cultivated crops and vegetables and to supply accurate information for management of resistance and prevent its outbreak in the future, we surveyed resistance to the newer chemistry compounds, as well as conventional insecticides against *S. litura* from various zones of the Hunan Province of China to ascertain whether or not the resistance was indeed evolving. This study is expected to be helpful in devising management strategies to overcome the resistance problems and to control *S. litura* under field conditions in the future.

## Materials and methods

### Insects

**Fig. 1:**
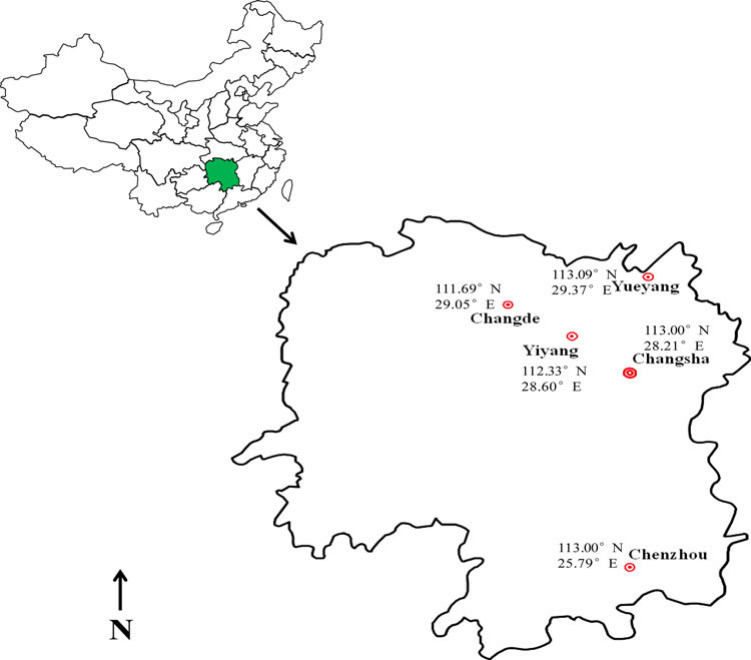
Sampling sites of *Spodoptera litura* in various zones of Hunan Province of China. The survey was carried out in the field season of 2010–2012. Surveyed province is highlighted in a *green shad*. (Color figure online)

A laboratory susceptible strain of *S. litura* was obtained from the Institute of Zoology, Beijing, China and designated as Lab-BJ. This strain was obtained by single pair crosses of a field-collected population of *S. litura* and reared in the laboratory for 6 years without exposure to insecticides. Bioassays were conducted in the laboratory to get the mortality data to use as a reference for baseline susceptibility of insecticides. Different populations of *S. litura* at fourth- or fifth-instar larvaes were collected from the field crops grown within a radius of almost 200 km of Hunan Province from 2010 to 2012. All strains were collected by walking through a 3 ha block of a particular crop in a zig-zag manner to get a mixed population from various areas (Fig. 1) and brought to the laboratory. The larvae were reared on semi-synthetic diet (Ahmad et al. 2008; Saleem et al. 2008) in the laboratory at 25 ± 3 °C and 65 ± 5 % RH in glass jars for at least two generations before the bioassays were carried out. Diet was replaced after 24 h and pupae were collected on alternate days. Moths were shifted to glass cages with mesh sides for ventilation and fed on a solution containing 10 % honey soaked onto cotton wool ball (Ahmad et al. 2007b). The neonate larvae were fed with semi-synthetic diet. The field-collected populations were reared in the laboratory to accommodate to laboratory conditions and to obtain sufficient insect numbers for bioassays.

### Insecticides

Ten insecticides were used in present study: 90 % emamectin benzoate (Hebei Veyong Bio-Chemical Co., Ltd., Hebei, China); 97.2 % abamectin (Hebei Veyong Bio-Chemical Co., Ltd., Hebei, China); 97 % indoxacarb (E.I. DuPont de Nemours and Co., Inc., Wilmington, DE, USA); 94.5 % chlorfenapyr (BASF (China) Co., Ltd., Beijing, China); 97.5 % chlorpyrifos (Jinan Luba Chemicals Co., Ltd., Jinan City, China); 91.6 % profenofos (Jiangsu Baoling Chemical Co., Ltd., Jiangsu, China); 95 % thiodicarb (Bayer CropScience Hangzhou Co., Ltd., Hangzhou, China); 98 % methomyl (Shandong Huayang Technology Co., Ltd., Ningyang, China); 98 % deltamethrin (Nanjing Redsun Co., Ltd., Nanjing, China); and 95 % bifenthrin (Bayer CropScience Hangzhou Co., Ltd., Hangzhou, China).

### Bioassays

Bioassays were conducted with newly third-instar larvae of *S. litura* using a standard leaf disk method (Sayyed et al. 2000; Ahmad et al. 2007b). Serial dilutions as mg/l of the active ingredient of the test compounds were prepared using 0.1 ‰ triton X-100 in water. Cotton leaves were cut into small, 9 cm pieces and dipped into the insecticide solution for 10 s. These leaves were air dried at ambient temperature for 5–10 min by spreading on a towel paper. Leaves were dipped in sterile distilled water and 0.1 ‰ triton X-100 only to use as controls. Leaves treated with insecticides were then transferred to each Petri dish lined with moistened filter paper. At least six concentrations and four replications (10 larvae per replication) were used to estimate each concentration mortality line thus total numbers of tested larvae per concentration were 40. The bioassays were kept at a temperature of 25 ± 3 °C, 65 ± 5 % relative humidity, and photoperiod of 16:8 (light: dark). Mortality data were scored 48 h after exposure for insecticides. Larvaes were considered dead if they failed to make a coordinated movement when prodded with a brush.

### Data analysis

Data obtained were corrected for control mortality using Abbott’s formula (1925) where necessary, and were analyzed by probit analysis through POLO-Plus (LeOra 2003) to estimate LC_50_ values and their 95 % fiducial limits (FLs). Resistance ratios (RRs) were determined as LC_50_ values of field strain/LC_50_ values of Lab-BJ. LC_50_ values were considered significantly different when they did not overlap with each other at their respective 95 % fiducial values (Litchfield and Wilcoxon 1949). The slope for regression line was compared with *t* test using SPSS software. A cross-resistance mechanism was determined among the tested insecticides by pairwise correlation coefficients of log LC_50_ values of the field populations by the Pearson correlation with the help of computer program XL-Stats.

## Results

### Toxicity of insecticides to laboratory strain

The results of bioassays for organophosphates against Lab-BJ showed that the profenofos was similar to the toxicity of chlorpyrifos based on the presence of overlap in the 95 % FLs, and among the carbamates tested, thiodicarb was less toxic than methomyl (*P* < 0.05), and bifenthrin was more toxic than deltamethrin in pyrethroids tested (*P* < 0.05) (Table 1). Among the newer chemistry insecticides tested, the most toxic was indoxacarb, and emamectin benzoate was the least toxic among the tested insecticides against the laboratory strain of *S. litura* (Table 1).

**Table 1:** Baseline susceptibilities of laboratory populations to ten insecticides

Insecticides	Toxic regression equation	LC_50_ (mg/l) (95 % FL)	Correlation coefficient
Chlorpyrifos	*Y* = 3.965 + 1.671*X*	4.18 (2.84–5.66)	0.9904
Profenofos	*Y* = 4.079 + 2.164*X*	3.75 (2.99–4.51)	0.9980
Thiodicarb	*Y* = 3.609 + 2.681*X*	6.42 (4.28–8.56)	0.9925
Methomyl	*Y* = 2.793 + 2.965*X*	1.28 (0.72–2.29)	0.9972
Deltamethrin	*Y* = 1.964 + 3.722*X*	3.99 (2.29–6.95)	0.9948
Bifenthrin	*Y* = 2.442 + 3.581*X*	0.51 (0.24–1.07)	0.9967
Emamectin	*Y* = 1.307 + 4.716*X*	0.67 (0.12–1.22)	0.9942
Abamectin	*Y* = 6.196 + 2.254*X*	0.28 (0.15–0.46)	0.9964
Indoxacarb	*Y* = 2.814 + 3.798*X*	0.08 (0.05–0.11)	0.9992
Chlorfenapyr	*Y* = 1.315 + 4.159*X*	0.54 (0.37–0.71)	0.9973

### Toxicity of conventional insecticides to field populations

In general, the RR for organophosphates ranged from 14- to 229-fold compared with the Lab-BJ population. The resistance to chlorpyrifos in *S. litura* was the lowest in a population collected from Changde, while the highest resistance was obtained in a population collected from Yiyang (Table 2). The resistance to chlorpyrifos ranged from 22-fold in the Changsha population to 120-fold in the Yiyang population in 2012. In the case of profenofos, *S. litura* in all five regions revealed higher resistance compared with chlorpyrifos tested, ranging from 24- (Changsha in 2010) to 229-fold (Changde in 2012) (Table 2). The average slope for the most of the field populations to organophosphates group was similar to the average slope of Lab-BJ population (Table 2).

**Table 2:** Toxicity of some organophosphates and synthetic pyrethroids insecticides against different populations of *Spodoptera litura* from Hunan, China

Insecticides	Location	Collected from	Collection data	Dose range (mg/l)	LC_50_ (mg/l) (95 % FL)	RR	Fit of probit line			*n*
							Slope (±SE)	*χ* ^2^	df	
Chlorpyrifos	Changsha	Taro	Sep-10	20–240	82.13 (42.37–120.23)	19.7	1.44 ± 0.32	1.26	4	240
		Cotton	Sep-11	25–300	103.37 (88.29–116.45)	24.7	1.61 ± 0.28	4.00	4	240
		Cotton	Oct-12	25–320	91.71 (46.37–137.02)	21.9	1.53 ± 0.22	1.76	4	240
	Yueyang	Lotus	Sep-10	75–800	307.06 (162.12–450.38)	73.5	1.26 ± 0.47	0.57	4	240
		Cotton	Aug-11	50–600	210.92 (138.38–292.37)	50.5	1.63 ± 0.24	0.39	4	240
		Lotus	July-12	65–800	262.55 (216.77–306.86)	62.8	1.72 ± 0.34	0.82	4	240
	Changde	Soybean	Aug-10	12–200	56.51 (30.27–82.76)	13.5	1.26 ± 0.17	1.14	4	240
		Cotton	Aug-11	30–480	176.81 (90.12–263.11)	42.3	0.67 ± 0.24	0.65	4	240
		Cotton	Oct-12	35–560	198.13 (95.4–296.86)	47.4	1.19 ± 0.21	2.74	4	240
	Yiyang	Soybean	Oct-10	50–800	230.32 (140.12–320.24)	55.1	1.31 ± 0.24	0.38	4	240
		Cotton	July-11	50–600	216.19 (110.12–322.23)	51.7	1.73 ± 0.25	0.41	4	240
		Cotton	Aug-12	100–1000	502.60 (366.65–636.17)	120	1.21 ± 0.61	0.26	4	240
	Chenzhou	Cotton	Oct-11	75–800	335.36 (263.47–405.36)	80.2	1.38 ± 0.25	0.38	4	240
		Sweet potato	Sep-12	75–800	285.62 (202.12–369.34)	68.3	1.14 ± 0.29	0.81	4	240
Profenofos	Changsha	Taro	Sep-10	25–300	90.6 (61.20–119.37)	24.2	1.48 ± 0.25	0.59	4	240
		Cotton	Sep-11	50–600	215.55 (153.75–275.55)	57.5	1.73 ± 0.29	3.04	4	240
		Cotton	Oct-12	60–900	276.86 (157.84–397.35)	73.8	1.67 ± 0.43	0.058	4	240
	Yueyang	Lotus	Sep-10	60–800	248.36 (142.33–354.68)	66.2	1.46 ± 0.36	0.68	4	240
		Cotton	Aug-11	75–900	392.81 (260.12–527.25)	105	2.01 ± 0.39	0.73	4	240
		Lotus	July-12	75–900	323.89 (200.23–446.68)	86.4	0.82 ± 0.26	0.38	4	240
	Changde	Soybean	Aug-10	70–1120	280.8 (176.59–384.58)	74.9	0.75 ± 0.27	1.59	4	240
		Cotton	Aug-11	80–1280	358.2 (213.47–500.96)	95.5	1.25 ± 0.17	1.34	4	240
		Cotton	Oct-12	150–2400	859.91 (644.87–1072.96)	229	1.90 ± 0.40	1.47	4	240
	Yiyang	Soybean	Oct-10	35–560	175.31 (112.13–237.43)	46.8	1.71 ± 0.14	0.29	4	240
		Cotton	July-11	45–720	198.15 (120.17–276.13)	52.8	0.92 ± 0.36	1.53	4	240
		Cotton	Aug-12	35–560	127.31 (64.50–192.13)	34.0	1.80 ± 0.27	0.81	4	240
	Chenzhou	Cotton	Oct-11	120–2000	462.98 (368.93–557.02)	123	1.57 ± 0.22	2.16	4	240
		Sweet potato	Sep-12	150–2400	631.65 (308.23–955.12)	168	1.84 ± 0.32	0.66	4	240
Thiodicarb	Changsha	Taro	Sep-10	62.5–1000	247.56 (140.21–354.63)	38.6	1.31 ± 0.23	1.69	4	240
		Cotton	Sep-11	375–6000	1762.87 (825.35–2769.39)	275	2.08 ± 0.39	0.80	4	240
		Cotton	Oct-12	375–6000	1657.13 (1210.28–2101.22)	258	2.02 ± 0.27	0.77	4	240
	Yueyang	Lotus	Sep-10	175–2800	734.38 (410.35–1057.44)	114	1.34 ± 0.15	1.83	3	240
		Cotton	Aug-11	125–2000	541.01 (253.83–826.20)	84.3	2.05 ± 0.24	0.17	4	240
		Lotus	July-12	200–3200	824.20 (623.18–1024.38)	128	2.11 ± 0.36	3.82	4	240
	Changde	Soybean	Aug-10	250–4000	1177.88 (732.16–1613.59)	183	2.60 ± 0.30	2.78	4	240
		Cotton	Aug-11	375–6000	1486.62 (731.92–2239.31)	232	1.53 ± 0.25	3.30	4	240
		Cotton	Oct-12	375–6000	1620.02 (1010.33–2228.56)	252	1.71 ± 0.24	0.79	4	240
	Yiyang	Soybean	Oct-10	350–5600	1404.18 (932.11–1873.25)	219	0.89 ± 0.26	0.60	4	240
		Cotton	July-11	450–7200	1786.94 (976.83–2583.06)	278	1.31 ± 0.24	1.98	4	240
		Cotton	Aug-12	625–10000	2725.87 (1214.17–4237.57)	425	2.47 ± 0.48	1.27	4	240
	Chenzhou	Cotton	Oct-11	175–2800	707.81 (342.37–1071.25)	110	1.78 ± 0.36	0.84	4	240
		Sweet potato	Sep-12	225–3600	952.54 (500.52–1406.13)	148	1.51 ± 0.66	2.45	4	240
Methomyl	Changsha	Taro	Sep-10	15–240	59.84 (34.17–85.51)	46.8	2.14 ± 0.24	0.99	4	240
		Cotton	Sep-11	150–2400	624.41 (420.24–826.33)	488	1.47 ± 0.55	0.39	4	240
		Cotton	Oct-12	200–3200	786.21 (317.26–1254.25)	614	1.37 ± 0.36	1.34	4	240
	Yueyang	Lotus	Sep-10	30–480	118.23 (72.35–164.12)	92.4	1.81 ± 0.18	0.76	4	240
		Cotton	Aug-11	70–1120	285.93 (180.72–389.93)	223	1.84 ± 0.19	1.50	4	240
		Lotus	July-12	62.5–1000	247.26 (124.38–489.75)	193	1.63 ± 0.28	0.28	4	240
	Changde	Soybean	Aug-10	50–800	215.76 (145.58–285.27)	169	2.01 ± 0.25	1.16	4	240
		Cotton	Aug-11	87.5–1400	350.95 (240.37–460.57)	274	2.22 ± 0.21	0.73	4	240
		Cotton	Oct-12	100–1600	400.96 (318.37–504.74)	313	2.26 ± 0.51	1.18	4	240
	Yiyang	Soybean	Oct-10	125–2000	469.44 (280.17–658.71)	367	2.15 ± 0.60	1.57	4	240
		Cotton	July-11	250–3000	1055.53 (738.74–1372.32)	825	1.73 ± 0.18	0.18	4	240
		Cotton	Aug-12	325–4000	1368.59 (628.31–2107.71)	1,069	2.23 ± 0.53	2.60	4	240
	Chenzhou	Cotton	Oct-11	20–320	87.40 (72.30–100.49)	68.3	1.94 ± 0.24	0.80	4	240
		Sweet potato	Sep-12	12.5–200	47.97 (23.25–72.70)	37.5	2.02 ± 0.45	0.74	4	240
Deltamethrin	Changsha	Taro	Sep-10	25–400	131.15 (93.57–168.73)	32.9	1.59 ± 0.18	0.42	4	240
		Cotton	Sep-11	112.5–1800	460.96 (300.26–620.87)	116	1.88 ± 0.21	1.33	4	240
		Cotton	Oct-12	37.5–2200	562.91 (304.55–1040.43)	141	1.53 ± 0.35	0.21	4	240
	Yueyang	Lotus	Sep-10	17.5–280	67.46 (43.53–90.60)	16.9	1.80 ± 0.37	0.20	4	240
		Cotton	Aug-11	25–400	142.12 (92.70–190.56)	35.6	1.57 ± 0.15	0.49	4	240
		Lotus	July-12	30–480	113.24 (72.13–154.34)	28.4	0.92 ± 0.28	1.41	4	240
	Changde	Soybean	Aug-10	37.5–600	156.21 (97.34–215.08)	39.2	1.91 ± 0.45	1.59	4	240
		Cotton	Aug-11	87.5–1400	367.60 (220.38–512.82)	92.1	0.82 ± 0.13	1.08	4	240
		Cotton	Oct-12	150–2400	622.74 (329.41–1177.26)	156	1.68 ± 0.32	0.83	4	240
	Yiyang	Soybean	Oct-10	17.5–280	69.35 (40.37–98.32)	17.4	1.13 ± 0.12	1.14	4	240
		Cotton	July-11	75–1200	333.05 (212.57–453.52)	83.5	0.92 ± 0.24	0.95	4	240
		Cotton	Aug-12	175–2800	693.52 (315.79–1523.06)	174	1.38 ± 0.25	1.61	4	240
	Chenzhou	Cotton	Oct-11	35–560	137.58 (76.39–197.46)	34.5	1.57 ± 0.15	1.23	4	240
		Sweet potato	Sep-12	75–1200	329.73 (152.63–505.77)	82.6	1.47 ± 0.42	0.62	4	240
Bifenthrin	Changsha	Taro	Sep-10	2.5–-30	8.80 (7.23–10.38)	17.3	1.55 ± 0.25	3.32	4	240
		Cotton	Sep-11	22.5–320	89.80 (71.85–107.74)	176	1.66 ± 0.46	1.98	4	240
		Cotton	Oct-12	25–400	116.02 (57.80–175.21)	227	1.73 ± 0.26	0.56	4	240
	Yueyang	Lotus	Sep-10	2–32	7.61 (6.25–8.98)	14.9	1.13 ± 0.29	0.60	4	240
		Cotton	Aug-11	7.5–120	29.82 (20.36–39.29)	58.5	2.33 ± 0.31	3.57	4	240
		Lotus	July-12	10–160	35.70 (19.82–64.30)	70.0	1.85 ± 0.22	0.49	4	240
	Changde	Soybean	Aug-10	1.5–24	6.28 (4.30–8.26)	12.3	1.99 ± 0.19	2.88	4	240
		Cotton	Aug-11	7.5–120	32.23 (21.14–43.32)	63.2	1.39 ± 0.26	1.08	4	240
		Cotton	Oct-12	7.5–120	28.46 (16.75–48.35)	55.8	3.43 ± 0.38	0.39	4	240
	Yiyang	Soybean	Oct-10	5–80	22.88 (16.59–29.17)	44.9	2.04 ± 0.20	1.36	4	240
		Cotton	July-11	5–80	19.17 (12.32–26.01)	37.6	1.73 ± 0.18	0.92	4	240
		Cotton	Aug-12	17.5–280	71.80 (42.80–120.45)	141	2.03 ± 0.37	0.56	4	240
	Chenzhou	Cotton	Oct-11	12.5–200	49.24 (34.69–63.79)	96.6	2.00 ± 0.29	1.76	4	240
		Sweet potato	Sep-12	15–240	62.40 (30.35–94.45)	122	1.76 ± 0.42	0.44	4	240

*RR* resistance ratio, calculated as LC_50_ of field/LC_50_ of Lab-BJ, *n* number of larvae used in bioassay, including controls, *χ* ^*2*^ values were not significant for all the assays

Two carbamates, thiodicarb, and methomyl tested in the present study, had very high levels of resistance, ranging from 38- to 1,069-fold compared with Lab-BJ strain. The resistance to methomyl was the highest in the population collected from Yiyang in 2012 with a RR of 1,069-fold while the lowest resistance (38-fold) from Chenzhou in 2012 (Table 2). On average, the RR was significantly higher in the populations collected from cotton, soybean, and lotus than the populations from taro and sweet potato (Table 2).

Among the pyrethroids, the RR ranged from 12- to 227-fold compared with the Lab-BJ population. The resistance to deltamethrin against *S. litura* was the lowest in a population collected from Yiyang in 2010, while the highest resistance to bifenthrin was obtained in a population collected from Changsha in 2012 (Table 2). In general, the average resistance level to deltamethrin and bifenthrin groups tested was equivalent. The average slope for the most of the field populations to pyrethroids group was similar to the average slope of Lab-BJ population; however, a substantial inter-population variation in slope was evident for bifenthrin, for example (3.43) for Changde population collected from cotton in 2012 (Table 2).

### Toxicity of newer chemistry insecticides to field populations

Results of the toxicity of newer chemistry insecticides i.e., emamectin benzoate, abamectin, indoxacarb, and chlorfenapyr against different populations are shown in Table 3. Emamectin benzoate is a synthetic analog of abamectin and the RR ranged from 1- to 22-fold when compared with the Lab-BJ strain. Almost all the populations collected from five districts for 3 years were significant resistance compared with Lab-BJ strain. The resistance to emamectin from Changsha, Yiyang, and Chenzhou populations showed increasing levels of resistance from 2010 to 2012.

**Table 3:** Toxicity of some newer insecticides against different populations of *Spodoptera litura* from Hunan, China

Insecticides	Location	Collected from	Collection data	Dose range (mg/l)	LC_50_ (mg/l) (95 % FL)	RR	Fit of probit line			*n*
							Slope (±SE)	*χ* ^2^	df	
Emamectin benzoate	Changsha	Taro	Sep-10	0.25–4	0.97 (0.15–1.79)	1.45	1.60 ± 0.19	0.61	4	240
		Cotton	Sep-11	0.5–8	2.40 (1.87–2.93)	3.58	2.07 ± 0.23	1.59	4	240
		Cotton	Oct-12	1.25–20	6.95 (4.83–9.07)	10.4	1.91 ± 0.22	2.60	4	240
	Yueyang	Lotus	Sep-10	0.25–4	1.51 (1.13–1.89)	2.25	2.18 ± 0.40	0.33	4	240
		Cotton	Aug-11	0.5–8	3.33 (2.75–3.91)	4.97	1.89 ± 0.36	0.85	4	240
		Lotus	July-12	0.5–8	3.24 (2.58–3.89)	4.83	1.58 ± 0.18	2.23	4	240
	Changde	Soybean	Aug-10	0.25–4	1.86 (1.55–2.18)	2.78	1.67 ± 0.19	1.38	4	240
		Cotton	Aug-11	1–16	5.24 (4.05–6.43)	7.82	1.55 ± 0.15	1.33	4	240
		Cotton	Oct-12	1–16	4.90 (3.67–6.22)	7.31	1.63 ± 0.28	0.76	4	240
	Yiyang	Soybean	Oct-10	0.5–8	3.44 (3.12–3.75)	5.13	2.02 ± 0.20	3.13	4	240
		Cotton	July-11	2.5–40	11.58 (7.38–15.79)	17.3	1.84 ± 0.29	0.72	4	240
		Cotton	Aug-12	1.25–20	6.29 (5.40–7.19)	22.5	1.35 ± 0.33	0.35	4	240
	Chenzhou	Cotton	Oct-11	1.25–20	5.82 (4.02–7.61)	8.68	1.69 ± 0.23	0.80	4	240
		Sweet potato	Sep-12	2–32	8.15 (3.93–12.36)	12.2	1.37 ± 0.35	0.54	4	240
Abamectin	Changsha	Taro	Sep-10	0.15–2.4	0.74 (0.53–0.95)	2.64	1.94 ± 0.24	2.01	4	240
		Cotton	Sep-11	0.5–8	2.42 (1.75–3.14)	8.64	1.80 ± 0.35	0.17	4	240
		Cotton	Oct-12	1.25–20	5.83 (4.35–7.31)	20.8	1.73 ± 0.16	0.79	4	240
	Yueyang	Lotus	Sep-10	1.875–30	10.55 (7.92–13.17)	15.7	1.83 ± 0.25	1.88	4	240
		Cotton	Aug-11	1.25–20	4.41 (3.29–5.52)	19.4	1.53 ± 0.34	0.54	4	240
		Lotus	July-12	1.875–30	9.69 (8.32–11.06)	34.6	2.21 ± 0.24	0.38	4	240
	Changde	Soybean	Aug-10	0.625–10	2.93 (2.16–3.71)	10.5	2.06 ± 0.37	2.22	4	240
		Cotton	Aug-11	0.75–12	4.34 (3.13–5.56)	15.5	1.94 ± 0.28	1.16	4	240
		Cotton	Oct-12	0.75–12	3.86 (3.09–4.61)	13.8	1.75 ± 0.35	0.49	4	240
	Yiyang	Soybean	Oct-10	0.25–4	1.72 (1.16–2.27)	6.13	2.20 ± 0.22	0.72	4	240
		Cotton	July-11	1–16	5.00 (3.75–6.24)	17.8	2.00 ± 0.26	1.30	4	240
		Cotton	Aug-12	1.25–20	6.29 (5.40–7.19)	22.5	1.35 ± 0.33	0.35	4	240
	Chenzhou	Cotton	Oct-11	1–16	5.07 (3.37–6.76)	18.1	1.69 ± 0.33	0.28	4	240
		Sweet potato	Sep-12	2.5–40	11.93 (7.27–16.59)	42.6	1.60 ± 0.41	0.59	4	240
Indoxacarb	Changsha	Taro	Sep-10	0.075–1.2	0.33 (0.26–0.41)	4.18	1.72 ± 0.27	2.60	4	240
		Cotton	Sep-11	0.2–3.2	0.80 (0.62–0.98)	10.0	1.47 ± 0.55	0.99	4	240
		Cotton	Oct-12	0.25–4	1.01 (0.72–1.30)	12.6	1.35 ± 0.33	0.35	4	240
	Yueyang	Lotus	Sep-10	0.05–0.8	0.23 (0.18–0.29)	2.92	1.75 ± 0.37	0.88	4	240
		Cotton	Aug-11	0.25–4	1.37 (1.15–1.60)	17.2	1.86 ± 0.35	1.66	4	240
		Lotus	July-12	0.5–8	1.77 (1.11–2.47)	22.1	1.51 ± 0.29	0.52	4	240
	Changde	Soybean	Aug-10	0.25–4	1.10 (0.88–1.31)	13.7	1.81 ± 0.29	0.18	4	240
		Cotton	Aug-11	0.5–8	1.79 (1.37–2.21)	22.4	2.24 ± 0.21	1.16	4	240
		Cotton	Oct-12	0.5–8	1.64 (0.98–2.38)	20.5	1.73 ± 0.35	0.41	4	240
	Yiyang	Soybean	Oct-10	0.025–0.4	0.15 (0.12–0.19)	1.92	1.95 ± 0.38	0.72	4	240
		Cotton	July-11	0.25–4	1.29 (0.83–1.74)	16.1	1.67 ± 0.27	1.58	4	240
		Cotton	Aug-12	0.25–4	1.15 (0.51–1.79)	14.4	1.58 ± 0.34	0.44	4	240
	Chenzhou	Cotton	Oct-11	0.05–0.8	0.29 (0.22–0.36)	3.65	1.48 ± 0.24	1.38	4	240
		Sweet potato	Sep-12	0.125–2	0.62 (0.311–0.921)	7.70	1.61 ± 0.37	0.62	4	240
Chlorfenapyr	Changsha	Taro	Sep-10	0.5–8	2.51 (1.97–3.04)	4.64	1.73 ± 0.16	2.01	4	240
		Cotton	Sep-11	0.5–8	2.10 (1.46–2.73)	3.88	1.57 ± 0.32	0.38	4	240
		Cotton	Oct-12	1.25–20	5.63 (3.05–10.40)	10.4	1.70 ± 0.35	0.11	4	240
	Yueyang	Lotus	Sep-10	0.625–10	2.58 (1.97–3.18)	4.77	1.94 ± 0.24	0.32	4	240
		Cotton	Aug-11	0.75–12	4.13 (3.16–5.09)	7.64	2.10 ± 0.33	0.45	4	240
		Lotus	July-12	0.75–12	3.52 (2.73–4.31)	6.52	1.81 ± 0.32	0.07	4	240
	Changde	Soybean	Aug-10	1.5–24	8.14 (6.32–9.97)	15.1	1.71 ± 0.24	1.71	4	240
		Cotton	Aug-11	1.5–24	9.72 (8.43–11.01)	18.0	1.36 ± 0.23	0.28	4	240
		Cotton	Oct-12	1.5–24	7.62 (3.77–15.41)	14.1	1.62 ± 0.36	0.40	4	240
	Yiyang	Soybean	Oct-10	1–16	4.43 (3.78–5.08)	8.20	1.83 ± 0.25	2.04	4	240
		Cotton	July-11	1.25–20	5.05 (3.92–6.18)	9.35	1.61 ± 0.56	0.26	4	240
		Cotton	Aug-12	2–32	8.86 (3.88–20.23)	16.4	2.17 ± 0.35	0.48	4	240
	Chenzhou	Cotton	Oct-11	1–16	4.41 (3.35–5.46)	8.16	1.78 ± 0.18	0.79	4	240
		Sweet potato	Sep-12	0.7–12	3.06 (1.63–4.48)	5.66	1.57 ± 0.31	1.01	4	240

*RR* resistance ratio, calculated as LC_50_ of field/LC_50_ of Lab-BJ, *n* number of larvae used in bioassay, including controls, *χ* ^*2*^ values was not significant for all the assays

Resistance level for abamectin ranged from 3- to 43-fold more than Lab-BJ strain. Populations collected from four locations, Changsha, Yueyang, Yiyang, and Chenzhou showed increasing trends in resistance levels, while Changde population in 2010–2012 showed varying levels of resistance. The population in 2012 from Chenzhou showed highest level of resistance with ratio of 43-fold compared to Lab-BJ, whereas the lowest level of tolerance was observed from Changsha district in 2010 with RR of threefold compared to Lab-BJ.

Out of 14 field populations tested for indoxacarb, 3 populations showed moderate level of resistance (21–22-fold), while other populations showed low level with a RR in the range of 2–17-fold was observed. The highest level of RR (22-fold) compared with Lab-BJ was observed from Changde in 2011 and Yueyang in 2012, whereas the lowest level of resistance (twofold) was recorded in a population from Yiyang (Table 3).

*Spodoptera litura* had exhibited low resistance to chlorfenapyr in general, with RRs commonly less than 20-fold compared with Lab-BJ (Table 3).The average slope for regression lines was similar for all five field populations except for Lab-BJ (Table 3).

### Pairwise correlations between log LC_50_ values of different insecticides

Correlation between the newer chemistry insecticides and old generation insecticides was not significant (*P* < 0.05) except abamectin, which was significant but negatively correlated with methomyl (Table 4). A significant correlation was observed between thiodicarb, methomyl, and deltamethrin (*P* < 0.01), whereas resistance to bifenthrin showed no correlations with resistance to other insecticides except deltamethrin (*P* < 0.05). There was lack of cross-resistance for emamectin, abamectin, indoxacarb, chlorfenapyr, chlorpyrifos, and profenofos in populations of *S. litura* from Hunan.

**Table 4:** Pairwise correlation coefficient comparison between log LC_50_ values of tested insecticides on field populations of *Spodoptera litura*

	Emamectin	Abamectin	Indoxacarb	Chlorfenapyr	Chlorpyrifos	Thiodicarb	Profenofos	Methomyl	Deltamethrin
Abamectin	0.280^ns^								
Indoxacarb	0.240^ns^	0.06^ns^							
Chlorfenapyr	0.235^ns^	−0.194^ns^	0.267^ns^						
Chlorpyrifos	0.301^ns^	0.516^ns^	−0.070^ns^	0.116^ns^					
Thiodicarb	0.460^ns^	−0.083^ns^	0.318^ns^	0.374^ns^	0.296^ns^				
Profenofos	0.207^ns^	0.287^ns^	0.306^ns^	0.155^ns^	0.054^ns^	−0.109^ns^			
Methomyl	0.517^ns^	−0.373^0.034^	0.282^ns^	0.400^ns^	0.304^ns^	0.289^ns^	−0.357^ns^		
Deltamethrin	0.446^ns^	−0.022^ns^	0.418^ns^	0.498^ns^	0.148^ns^	0.804^0.001^	0.236^ns^	0.683^0.007^	
Bifenthrin	0.322^ns^	0.146^ns^	0.047^ns^	−0.017^ns^	0.066^ns^	0.474^ns^	0.022^ns^	0.432^ns^	0.639^0.014^

Superscripts represent significance of the regression

## Discussion

The present study, conducted from 2010 to 2012, demonstrate that the *S. litura* populations on five cash crops in five regions of Hunan Province have shown varying degrees of resistance to six conventional insecticides and four newer insecticides. This suggests that populations of *S. litura* have the potential to develop resistance to a wide range of chemicals.

The resistance to organophosphates, which act as acetylcholinesterase inhibitors (Ahmad et al. 2007a, b), was found at a high level (>50-fold) in most of populations except the populations collected from Changsha and Changde, which was medium level (20–50-fold) resistance to chlorpyrifos (Table 2). This could be related to the commonly reliance in the use of organophosphates against insects in these areas. Resistance in *S. litura* against organophosphates has been reported from various parts of the Asia countries, such as Pakistan (Ahmad et al. 2007a, b; Saleem et al. 2008; Shad et al. 2012), India (Armes et al. 1997; Kranthi et al. 2002), and China (Huang et al. 2006). There were also reports of resistance development in beet armyworm *Spodoptera exigua* (H.), a species closely related to *S. litura*, from Guatemala (Delorme et al. 1988), Mexico (Teran-Vargas et al. 1997), Nicaragua (Pérez et al. 2000), Pakistan (Ahmad and Arif 2010; Ishtiaq et al. 2012), and China (Mu et al. 2005; Zhou et al. 2011), providing evidence of high level of resistance against organophosphates insecticides. As carbamates were more effective insecticide against lepidopteran pests, including *Spodoptera* spp. (Ahmad et al. 2008; Saleem et al. 2008; Shad et al. 2012), the application of this insecticide group was widely used to control *S. litura* in recent years. In most areas of Hunan, farmers used carbamates more than five times a month, so all the populations showed very high level of resistance except the population from taro, which showed moderate level of resistance against carbamates, as taro was sporadic cultivation and insecticide was seldom for use in such vegetable. The resistance to synthetic pyrethroids (deltamethrin and bifenthrin) was found at high or very high level in all populations collected from Hunan in 2012 except the deltamethrin population from Yueyang, as this insecticide was forbidden for use in vegetables for export in Yueyang. The tendency of increasing resistance to pyrethroids is consistent with the results of Huang et al. (2006) and Xie et al. (2010), and this could be related to the increase in the use of pyrethroids in these areas.

Although variation in susceptibility to laboratory strain was observed among the newer insecticides tested, the magnitude of the differences was small, less than ninefold for these four newer insecticides (Table 1). These results suggest that the observed susceptibility differences reflect natural variation in laboratory strain susceptibility among the newer insecticides rather than variation caused by prior exposure to selection pressure. Overall, the laboratory strain was relatively more sensitive than the field populations, particularly to indoxacarb (Table 1). Different members of newer chemistry insecticides exhibited different levels of toxicity, which will be helpful in devising management strategies. Emamectin benzoate and abamectin belong to the avermectins group and act as chloride channel activators (Teran-Vargas et al. 1997). Emamectin looked to be an effective insecticide because it exhibited low level of resistance in most of the populations tested. Therefore, emamectin is still considered as an effective tool for management of *S. litura* for most of the areas. Indoxacarb acts as a voltage-dependent sodium channel blocker belonging to the oxadiazine insecticide group (Sayyed et al. 2008), and chlorfenapyr has a novel mode of action, targeting oxidative pathways in insect mitochondria (Van Leeuwen et al. 2006). Indoxacarb and chlorfenapyr exhibited low level of resistance in all populations tested except only one medium resistance population, suggesting its effectiveness for *S. litura* management for most of the areas. The low application of newer insecticides is also associated with their high price, which many farmers could not afford. However, this cannot explain why abamectin resulted in higher resistance compared with other newer insecticides in most of the populations in 2012, and pairwise comparisons of the log LC_50_ values of insecticides tested showed occurrence of correlation within abamectin and methomyl (Table 4), which suggest that resistance to abamectin might due to a possible cross-resistance mechanism to conventional insecticides. A significant higher correlation between abamectin and emamectin benzoate has been reported from *S. litura* in Pakistan (Ahmad et al. 2008), our papers do not derive this results, although abamectin and emamectin both bind to the GABA-gated chloride channel. Previous studies reported that the detoxification enhancement causes metabolism resistance and involves different enzymes, including cytochrome P450 monooxygenase (MFO), carboxylesterase and esterase (Ishaaya and Casida 1980; Scott 1999; Huang et al. 2006; Chen et al. 2008) and both MFO and esterase have many isoenzymes which all have a range of substrates. If an insecticide selects specific isoenzymes, which can act on different insecticides, cross-resistance might be possible. Maybe the significant correlation between abamectin and methomyl is that methomyl has specific isoenzymes that associated with the abamectin. Resistance to newer chemistry insecticides in *S. litura* has not yet been reported from cash crops growing areas of Hunan, China to the best of our knowledge, except one reported paper in which they have identified resistance in *S. litura* from two locations in Jiangsu and Anhui Provinces (Huang et al. 2006). Insecticide resistance is an increasing concern in agricultural crops of China against almost all the major insect pests such as cotton bollworm *Helicoverpa armigera* (H.) (Wu et al. 2005; Wu 2007), sweet potato whitefly *Bemisia tabaci* (Gennadius) (Luo et al. 2010; Wang et al. 2010a, b), diamondback moth *Plutella xylostella* (L.) (Zhao et al. 2006; Wang et al. 2010a, b), western flower thrips *Frankliniella occidentalis* (Pergande) (Chen et al. 2011), and beet armyworm *S. exigua* (H.) (Mu et al. 2005; Zhou et al. 2011). These insects have been reported to develop resistance either against different groups or the representative of some group of insecticides. On the other hand, illiteracy can be one of the reasons for indiscriminate insecticides use for the development of insecticidal resistance in the most of major pests of cash crops.

*Spodoptera litura* has recently emerged as a serious pest of cash crops in Hunan, China. The development of a broad-spectrum resistance to insecticides has complicated its chemical control. However, the control of *S. litura* has relied mainly on the application of various insecticides. It is very important to select several effective insecticides to control this pest. The successful management of insecticide resistance depends ultimately on a thorough knowledge of its genetic basis and the mechanisms involved. The mode of inheritance helps in resistance detection, monitoring, modeling and risk assessment. Such knowledge can provide the basis for management programs aimed at minimizing the development of resistance. From the results of this article, we propose newer and conventional insecticides, which have different resistance mechanisms as effective insecticides rotation program for *S. litura* in Hunan. In order to protect those insecticides and postpone the development of resistance, a resistance management strategy of decreased selection pressure could be achieved by alternations these insecticides on basis of proper pest scouting and pest status for decision of control application or using insecticides when economic injury levels are achieved. Alternative pest management practices, such as cultural, pheromones traps, parasitoids, and predators could also help to reduce the selection pressure. Prognosis on the basis of light or pheromone-traps and prevailing meteorological conditions may help in determining better timing of control operations. Slow-release pheromone formulations have shown success for mating disruption (Wei and Du 2004). It could also help to conserve the parasitoids of *S. litura* or microbial parasites such as nucleopolyhedrovirus (Nathan and Kalaivani 2005; Nguyen et al. 2005), which is necessary to reduce pesticide applications. *Bacillus thuringiensis* toxins (Cry1Ca and Cry1F) which are also effective against *S. litura* (Zhang et al. 2006) and other major insect pests such as *H. armigera* (Wan et al. 2005; Wu et al. 2008), stacking them in a crop plant and using as an integrated pest management tool could also be another promising management strategy.

## References

[CR1] Abbott WS (1925). A method of computing the effectiveness of an insecticide. J Econ Entomol.

[CR2] Ahmad M, Arif MI (2010). Resistance of beet armyworm *Spodoptera exigua* (Lepidoptera: Noctuidae) to endosulfan, organophosphorus and pyrethroid insecticides in Pakistan. Crop Prot.

[CR3] Ahmad M, Arif MI, Ahmad M (2007). Occurrence of insecticide resistance in field populations of *Spodoptera litura* (Lepidoptera: Noctuidae) in Pakistan. Crop Prot.

[CR4] Ahmad M, Sayyed AH, Crickmore N, Saleem MA (2007). Genetics and mechanism of resistance to deltamethrin in a field population of *Spodoptera litura* (Lepidoptera: Noctuidae). Pest Manag Sci.

[CR5] Ahmad M, Sayyed AH, Saleem MA (2008). Evidence for field evolved resistance to newer insecticides in *Spodoptera litura* (Lepidoptera: Noctuidae) from Pakistan. Crop Prot.

[CR6] Armes NJ, Wightman JA, Jadhav DR, Ranga Rao GV (1997). Status of insecticide resistance in *Spodoptera litura* in Andhra Pradesh, India. Pestic Sci.

[CR7] Bisset J, Rodriguez M, Soca A, Pasteur N, Raymond M (1997). Cross-resistance to pyrethroid and organophosphorus insecticides in the southern house mosquito (Diptera: Culicidae) from Cuba. J Med Entomol.

[CR8] Chen Q, Jin QA, Peng ZQ, Tang C, Wen HB (2008). Analysis of the susceptibility of *Spodoptera litura* (Fabricius) to abamectin. Chin Agric Sci Bull.

[CR9] Chen XL, Yuan LZ, Du YZ, Zhang YJ, Wang JJ (2011). Cross-resistance and biochemical mechanisms of abamectin resistance in the western flower thrips, *Frankliniella occidentalis*. Pestic Biochem Physiol.

[CR47] Delorme R, Fournier D, Chaufaux J, Cuany A, Bride JM, Auge D, Berge JB (1988) Esterase metabolism and reduced penetration are causes of resistance to deltamethrin in *Spodoptera exigua* HUB (Noctuidea; lepidoptera). Pestic Biochem Phys 32:240–246

[CR10] Huang SJ, Xu JF, Han ZJ (2006). Baseline toxicity data of insecticides against the common cutworm *Spodoptera litura* (Fabricius) and a comparison of resistance monitoring methods. Int J Pest Manag.

[CR11] Ishaaya I, Casida JE (1980). Properties and toxicological significance of esterases hydrolyzing permethrin and cypermethrin in *Trichoplusia ni* larval gut and integument. Pestic Biochem Physiol.

[CR12] Ishtiaq M, Saleem MA, Razaq M (2012). Monitoring of resistance in *Spodoptera exigua* (Lepidoptera: Noctuidae) from four districts of the Southern Punjab, Pakistan to four conventional and six new chemistry insecticides. Crop Prot.

[CR13] Kranthi KR, Jadhav DR, Wanjari RR, Ali SS, Russell D (2001). Carbamate and organophosphate resistance in cotton pests in India, 1995 to 1999. Bull Entomol Res.

[CR14] Kranthi KR, Jadhav DR, Kranthi S, Wanjari RR, Ali SS, Russell DA (2002). Insecticide resistance in five major insect pests of cotton in India. Crop Prot.

[CR15] LeOra S (2003). Poloplus, a user’s guide to probit and logit analysis.

[CR16] Litchfield JT, Wilcoxon FA (1949). A simplified method of evaluating dose–effect experiments. J Pharmacol Exp Ther.

[CR17] Luo C, Jones CM, Devine G, Zhang F, Denholm I, Goman K (2010). Insecticide resistance in *Bemisia tabaci* biotype Q (Hemiptera: Aleyrodidae) from China. Crop Prot.

[CR18] Matsuura H, Naito A (1997). Studies on the cold-hardiness and overwintering of *Spodoptera litura* F. (Lepidoptera: Noctuidae): VI. Possible overwintering areas predicted from meteorological data in Japan. Appl Entomol Zool.

[CR19] Mu W, Wu KM, Zhang WJ, Guo YY (2005). Cross-resistance and relative fitness of lambda-cyhalothrin resistant near-isogenic lines in *Spodoptera exigua* (Hübner). Sci Agric Sin.

[CR20] Nathan SS, Kalaivani K (2005). Efficacy of nucleopolyhedrovirus and azadirachtin on *Spodoptera litura* Fabricius (Lepidoptera: Noctuidae). Biol Control.

[CR21] Nguyen DH, Nakai M, Takatsuka J, Okuno S, Ishii T, Kunimi Y (2005). Interaction between a nucleopolyhedrovirus and the braconid parasitoid *Meteorus pulchricornis* (Hymenoptera: Braconidae) in the larvae of *Spodoptera litura* (Lepidoptera: Noctuidae). Appl Entomol Zool.

[CR22] Pérez CJ, Alvarado P, Narváez C, Miranda F, Hernández L, Vanegas H, Hruska A, Shelton AM (2000). Assessment of insecticide resistance in five insect pests attacking field and vegetable crops in Nicaragua. J Econ Entomol.

[CR23] Raymond M, Marquine M (1994). Evolution of insecticide resistance in *Culex pipiens* populations: the Corsican paradox. J Evol Biol.

[CR24] Sahayaraj K, Paulraj MG (1998). Screening the relative toxicity of some plant extracts to *Spodoptera litura* Fab. (Insecta: Lepidoptera: Noctuidae) of groundnut. Fresenius Environ Bull.

[CR25] Saleem MA, Ahmad M, Aslam M, Sayyed AH (2008). Resistance to selected organochlorin, organophosphate, carbamate and pyrethroid, in *Spodoptera litura* (Lepidoptera: Noctuidae) from Pakistan. J Econ Entomol.

[CR26] Sayyed AH, Haward R, Herrero S, Ferre J, Wright DJ (2000). Genetic and biochemical approach for characterization of resistance to *Bacillus thuringiensis* toxin Cry1Ac in a field population of the diamondback moth, *Plutella xylostella*. Appl Environ Microbiol.

[CR27] Sayyed AH, Saeed S, Noor-ul-ane M, Crickmore N (2008). Genetic, biochemical, and physiological characterization of spinosad resistance in *Plutella xylostella* (Lepidoptera: Plutellidae). J Econ Entomol.

[CR28] Scott JG (1999). Cytochromes P450 and insecticide resistance. Insect Biochem Mol Biol.

[CR29] Shad SA, Sayyed AH, Fazal S, Saleem MA, Zaka SM, Ali M (2012). Field evolved resistance to carbamates, organophosphates, pyrethroids, and new chemistry insecticides in *Spodoptera litura* Fab. (Lepidoptera: Noctuidae). J Pest Sci.

[CR30] Su JY, Lai TC, Li J (2012). Susceptibility of field populations of *Spodoptera litura* (Fabricius) (Lepidoptera: Noctuidae) in China to chlorantraniliprole and the activities of detoxification enzymes. Crop Prot.

[CR31] Teran-Vargas AP, Garza-Urbina E, Blanco-Montero CA, Perez-Carmona G, Pellegaud-Rabago JM (1997) Efficacy of new insecticides to control beet armyworm in north eastern Mexico. In: Proceedings of the Beltwide Cotton Conference of the National Cotton Council, New Orleans, Louisiana, pp. 1030–1031

[CR32] Torres-Vila LM, Rodriguez-Molina MC, Lacasa-Plasencia A, Bielza-Lino P (2002). Insecticide resistance of *Helicoverpa armigera* to endosulfan, carbamates and organophosphates: the Spanish case. Crop Prot.

[CR33] Van Leeuwen TV, Pottelberge SV, Tirry L (2006). Biochemical analysis of a chlorfenapyr-selected resistant strain of *Tetranychus urticae* Koch. Pest Manag Sci.

[CR34] Wan P, Zhang YJ, Wu KM, Huang MS (2005). Seasonal expression profiles of insecticidal protein and control efficacy against *Helicoverpa armigera* for Bt cotton in the Yangtze River Valley of China. J Econ Entomol.

[CR35] Wang JJ, Dong HG, Yuan LZ (2009). Resistance mechanisms of *Spodoptera litura* to indoxacarb. Acta Phytophysiol Sin.

[CR36] Wang XL, Li XY, Shen AD, Wu YD (2010). Baseline Susceptibility of the diamondback moth (Lepidoptera: Plutellidae) to chlorantraniliprole in China. J Econ Entomol.

[CR37] Wang ZY, Yan HF, Yang YH, Wu YD (2010). Biotype and insecticide resistance status of the whitefly *Bemisia tabaci* from China. Pest Manag Sci.

[CR38] Wei HY, Du JW (2004). Sublethal effects of larval treatment with deltamethrin on moth sex pheromone communication system of the Asian corn borer, *Ostrinia furnacalis*. Pestic Biochem Physiol.

[CR39] Wu KM (2007). Monitoring and management strategy for *Helicoverpa armigera* resistance to Bt cotton in China. J Invertebr Pathol.

[CR40] Wu SC, Gu YZ, Wang DS (1995). Resistance of the tobacco army moth (*Prodenia litura*) to insecticides and its control. Acta Agric Shanghai.

[CR41] Wu KM, Mu W, Liang GM, Guo YY (2005). Regional reversion of insecticide resistance in *Helicoverpa armigera* (Lepidoptera: Noctuidae) is associated with the use of Bt cotton in northern China. Pest Manag Sci.

[CR42] Wu KM, Lu YH, Feng HQ, Jiang YY, Zhao JZ (2008). Suppression of cotton bollworm in multiple crops in China in areas with Bt toxin-containing cotton. Science.

[CR43] Xie SH, Liang YP, Lin ZF, Li H, Ji XC (2010). The toxicity and control efficiency of 9 insecticides to *Spodoptera litura*. Plant Prot.

[CR44] Zhang GF, Wan FH, Liu WX, Guo JY (2006). Early instar response to plant-delivered Bt-toxin in a herbivore (*Spodoptera litura*) and a predator (*Propylaea japonica*). Crop Prot.

[CR45] Zhao JZ, Collins HL, Li YX, Mau RFL, Thompson GD, Hertlein M, Andaloro JT, Boykin R, Shelton AM (2006). Monitoring of diamondback moth (Lepidoptera: Plutellidae) resistance to spinosad, indoxacarb, and emamectin benzoate. J Econ Entomol.

[CR46] Zhou C, Liu YQ, Yu WL, Deng ZR, Gao M, Liu F, Mu W (2011). Resistance of *Spodoptera exigua* to ten insecticides in Shandong, China. Phytoparasitica.

